# Hepatitis C Virus as a Multifaceted Disease: A Simple and Updated Approach for Extrahepatic Manifestations of Hepatitis C Virus Infection

**Published:** 2010-12-01

**Authors:** Mahmoud Aboelneen Khattab, Mohammed Eslam, Seyed Moayed Alavian

**Affiliations:** 1 Department of Internal Medicine, Minia University, Minia, Egypt; 2 Baqiyatallah Research Center for Gastroenterology and Liver Disease, Baqiyatallah University of Medical Sciences, Tehran, IR.Iran

**Keywords:** Hepatitis C virus, public health, Disease

## Abstract

Hepatitis C virus infection is an emerging disease and  a public health problem in the world. There are accumulating data regarding extra hepatic manifestation of HCV, such as rheumatologic manifestations, endocrine, hematologic, dermatologic, renal, neurologic, and systemic manifestations. The therapy of them needs more attention to some exacerbations of extra hepatic manifestation and in some situation it needs different approaches. In this review we tried to provide latest evidence for extra hepatic manifestation and management of them.

## Introduction

Hepatitis C virus (HCV) is a major cause of liver-related morbidity and mortality worldwide and represents a major public health problem [1][2][3][4][5][6]. HCV can spread parenterally through contact with infected blood, transfusion of infected blood and its products, intravenous drug use, contamination during medical procedures, and a lack of attention to health precautions. Despite a declining incidence of new infections [7][8], the burden of the disease, both in terms of mortality and cost, is expected to increase over the next decade, and HCV infection will maintain to be a potential cause of morbidity and mortality and need for transplantation in the future [9][10]. It is estimated that around 170 to 200 million individuals are living with HCV infection worldwide [11][12], and there is significant geographical variation in the prevalence of HCV infection across countries and regions [1][13]. Although HCV is a hepatotropic virus, in some patients the primary manifestations of infection occur outside the liver. There is a growing body of evidence to support the idea that HCV can replicate efficiently in extrahepatic tissues including the PBMC. Autoimmune manifestations are common in patients chronically infected by HCV [14]. These manifestations can be dominant, whereas the hepatic disease can be quiescent or mild. More recently, there has been growing interest in the relationship between HCV and Sjogren's syndrome (SS), rheumatoid arthritis (RA), and systemic lupus erythematosus (SLE(15). Depending on the pathogenic and epidemiological evidence provided by different studies; the extrahepatic manifestations of HCV infection (EHMs-HCV) can be classified into four categories:

1.  EHMs-HCV characterized by a very strong association demonstrated by both epidemiological and pathogenetic evidence (e.g., mixed cryoglobulinemia);

2.  EHMs-HCV include disorders for which the significant association with HCV infection is supported by enough data to clearly show a higher prevalence of HCV than in controls but still have unclear pathogenic mechanisms (e.g., B-cell-derived non-Hodgkin's lymphoma [NHL], diabetes mellitus, porphyria cutanea tarda, lichen planus);

3.  EHMs-HCV includes the associations for which the high prevalence in HCV populations could be due to HCV infection or confounding factors, and thus these associations still require confirmation and a more detailed characterization with respect to similar pathologies of different etiology or idiopathic nature (e.g., idiopathic pulmonary fibrosis, autoimmune thyroiditis, sicca syndrome, noncryoglobulinaemic nephropathies and glomerulonephritis, and aortic atherosclerosis);

4.  EHMs-HCV includes only anecdotal observations (e.g., growth hormone defficiency, chronic pruritus, cardiomyopathy, psoriasis, peripheral or central neuropathies, chronic polyarthritis, rheumatoid arthritis, polyarthritis nodosa, behcet's syndrome, poly or dermatomyositis, necrolytic acral erythema, and autoimmune hemolytic anemia).

## 1. Mixed Cryoglobulinemia

Mixed Cryoglobulinemia (MC) is the most documented and closely associated disorder with HCV [16][17]. The prevalence of HCV-infected patients with coexisting circulating MC ranges from less than 10% to greater than 50%; however, overt vasculitis manifestations are seen in only 2% to 3% of these patients [18][19][20]. This variability may represent geographic and population-specific factors involved in the development of MC, differences in the definition of the disease, and laboratory techniques for diagnosis. The disease occurs as a result of chronic immune-system stimulation leading to B-cell clonal expansion and immune-complex (IgG, IgM, RF complement, HCV-LDL/VLDL) production. These immune complexes will often take the form of cryoglobulins [21][22][23]. Cryoglobulins are monoclonal or polyclonal immunoglobulins that reversibly precipitate at low temperatures; cryoglobulinemia occurs when these proteins are present in the circulation [24]. Clinical manifestations of MC are secondary to a systemic immune-complex-related vasculitis involving small vessels.

### Diagnosis of Cryoglobulinemia

Nowadays, there are no standardized criteria for the diagnosis of MCS. However, valuable classifications have been proposed by the Italian Group for the Study of Cryoglobulinemia [24]. Diagnosis is based on clinicopathological and laboratory findings. Cryoglobulinemia may be suspected if the patient has positive rheumatoid factors. Clinically, asymptomatic serum MC can be found in some individuals chronically infected with HCV [24][25]; a condition that may precede the clinical onset of the disease by years or decades. Glomerulonephritis, peripheral neuropathy, and generalized vasculitis are the common complications of cryoglobulinemia [26][27][28].

**Figure 1:**
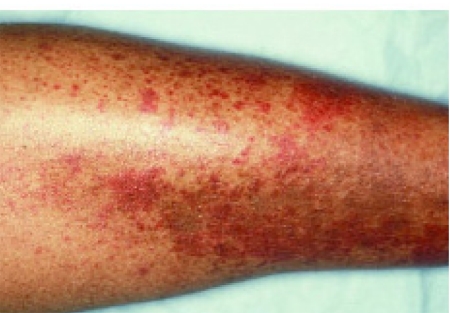
Nonblanching erythematosus papules are the notable findings of these palpable purpura, which are characteristic of the vasculitis associated with MC.

Palpable purpura (Figure. 1) is the most common clinical finding, occurring in 90% of cases. The association between MC and severe liver damage or steatosis has been discussed widely [29][30][31]. Several studies have shown an epidemiological association between MC and severe liver damage [29]. However, the pathogenetic mechanisms of such an association have not been clearly identified. The laboratory work-up of cryoglobulinemia vasculitis includes cryoglobulin testing, quantification of total serum protein and immunoglobulins, complement levels, evaluation of serum for monoclonal gammopathy, RF activity, virological markers (anti-HCV antibodies, HCV RNA, hepatitis B virus serology, hepatitis B virus DNA, and others), blood chemistry, and urine analysis. Leukocytoclastic vasculitis, involving medium- and, more often, small-sized blood vessels (arterioles, capillaries, and venules) is the typical pathological finding of involved tissues. Leukocytoclastic vasculitis is easily detectable by means of skin biopsy of recent vasculitis lesions (within the first 24 to 48 hours [24][32][33].

### Treatment options

Treatment can eradicate HCV infection (etiologic therapy), suppress B-cell clonal expansion and cryoglobulin production (pathogenetic therapy), or ameliorate symptoms (symptomatic therapy).

### Etiologic therapy

Under ideal circumstances, the treatment of MC aims to eradicate the HCV infection. Treatment of MC with interferon (IFN) therapy is associated with a relatively poor response [34][35]; however, PEG-IFN plus RBV show better results [36][37]. The goal of therapy in those patients is not limited to a sustained virologic response; rather, patients might see an improvement in their renal manifestations with prolonged treatment courses. However, clinical improvements are often transient and restricted to patients with mild to moderate disease activity [36][37][38]. Moreover, IFN therapy can induce an exacerbation of various vasculitis manifestations (i.e., glomerulonephritis, neuropathy), and RBV, due to its renal elimination, may be contraindicated in patients with severe renal impairment.

### Pathogenetic therapy

This therapy is currently used when antiviral therapy is not recommended. Treatment should be limited to the time (weeks or months) required for symptom remission. Several alternative therapies may be adopted, which include corticosteroids due to its anti-inflammatory and immunosuppressive actions (at high doses: 1 mg/kg daily or 0.5-1 g). However, the disadvantage of favoring the etiologic agent is that it can lead to increased viral replication [39]. Immunosuppressive drugs (e.g., Cyclophosphamide, Chlorambucil, and Azathioprine) are used to suppress antibody and cryoglobulin production [40][41]. The most effective and commonly used cytotoxic drug is Cyclophosphamide, given orally at doses of 2 mg/kg per day. Recently; Mycophenolate Mofetil (1 g twice a day) can be used as a less toxic alternative to Cyclophosphamide for the induction of remission in MC vasculitis; Mycophenolic Acid is more selective than Cyclophosphamide in inhibiting lymphocyte proliferation and functions. Interestingly, Mycophenolic Acid seems to reduce viremia in HCV-infected renal or heart-transplant recipients due to its ability to inhibit inosine monophosphate dehydrogenase, the same target enzyme inhibited by RBV [42]. However, data supporting this approach are limited and almost exclusively derived from anecdotal reports [43]. For patients unresponsive to treatment with steroids or other immunosuppressants, the administration of the novel immunosuppressant Rituximab; a chimeric monoclonal antibody directed against CD20 antigen on B cells, has been recently proposed for the pathogenetic treatment of HCV-related MC [44][45]. By depleting B cells, Rituximab has the potential to reduce the development of plasma cells, thereby limiting Cryoglobulin production. Rituximab, at the standard dose of 375 mg/m2 weekly for 4 weeks proved to be a safe and effective treatment for most patients with HCV-MC, leading to significant clinical improvement as a consequence of both B-cell depletion and decreases in serum Cryoglobulin levels [44][45][46][47][48]. Although fever, chills, nausea, vomiting, urticaria, orthostatic hypotension, and bronchospasm occur in more than 80% of patients, these side effects are generally mild and limited to the infusion period. An increase in viral load, without significant variations in liver-function tests, has been detected after rituximab treatment [44] To reduce HCV replication, a combination of Rituximab with antiviral agents has been suggested.

Because the median duration of the response to Rituximab therapy is about 1 year, a relapse of cryoglobulinemic vasculitis may develop following treatment. Relapses are preceded by peripheral B-cell repletion. It is unknown whether maintenance therapy with Rituximab is better than retreatment after relapse. Plasmapheresis can be used as an effective adjuvant therapy to treat severe exacerbations of cryoglobulinemic vasculitis, particularly active cryoglobulinemic glomerulonephritis. Both traditional plasma exchange and double-filtration plasma exchange are able to markedly reduce the levels of circulating immune complex, especially the cryoglobulins. Oral Cyclophosphamide (50 to 100 mg/day for 2 to 6 weeks) during the tapering of apheretic sessions can reinforce the beneficial effect of plasma exchange; moreover, it can prevent the rebound phenomenon that may be observed after the discontinuation of aphaeresis [33].

### Symptomatic Therapy

The hypoantigenic diet (LAC) diet consists of a diet with reduced content of alimentary macromolecules with high antigenic properties, allowing for more efficient removal of CGs by the reticuloendothelial system. This diet can improve minor manifestations of the disease (purpura, arthralgias, paresthesias) and is generally prescribed at the initial stage of the disease [49]. Colchicine, an anti-inflammatory agent with a relatively selective effect for gouty arthritis, has been proposed for MC patients with mild to moderate levels of the disease. In an uncontrolled trial, colchicine (1 mg/day for 6 to 48 months) improved clinical and laboratory variables (particularly cryocrit; [50]. However, its toxicity and the availability of alternative, less toxic agents have substantially lessened its use.

## 2. Lymphoproliferative disorders (LPD)

HCV-associated LPD can be observed during the course of MC or in non-MC-related idiopathic forms [51]. A recent, large-scale, retrospective cohort study suggests that HCV infection confers a 20-30% increased risk of NHL overall [52]. These results were confirmed in a meta-analysis by Dal Maso and Franceschi, which included 15 case-controlled studies and 3 cohort studies and demonstrated a pooled risk ratio (relative risk [RR]) of 2.0 (95% CI, 1.8 to .2) for the cohort studies and 2.5 (95% CI, 2.1 to 3.1) for the case-controlled studies for the development of B-cell NHL in HCV-infected patients [53]. The meta-analysis did not show differing RRs for NHL subtypes. From a histopathological point of view, although virtually all types of lymphoid malignancy can be found in patients with HCV infection, the strongest association is with NHL, and the vast majority of NHL is low grade with predominantly extranodal involvement. According to the REAL/WHO classifications, the most prevalent HCV-associated LPDs are follicular lymphoma, B-cell chronic lymphocytic leukemia or small lymphocyte lymphoma, diffuse large B-cell lymphoma, and marginal zone lymphoma, including the mucosa-associated lymphoid tissue lymphoma [54]. Overall, marginal-zone lymphoma appears to be the most frequently encountered low-grade B-cell lymphoma in HCV patients [55]. Regarding the pathogenesis of the HCV-associated LPDs, the majority of studies pinpoint two presumable mechanisms. The first is indirect; specifically, by relying on the chronic nature of HCV infection, chronic antigenic stimulation may lead to an overexpression of B-cells favoring certain clones. This mechanism could explain the immune dysregulation leading to autoimmunity, MC, and eventually malignant transformation [56]. The second mechanism is direct, relying on the particular lymphotropism of HCV and therefore on the high invasion of B-cells by HCV [57]. Both mechanisms lead simultaneously through complex, multistep, pathogenic pathways. There may be genetic and environmental factors that further explain the final steps to malignant transformation. Rearrangement of the antiapoptotic bcl-2 gene with t(14;18) translocation is the most common chromosomal translocation in lymphoid cancers, especially follicular lymphoma, a subtype of NHL. Thirty-five percent of patients with chronic HCV infection have evidence of the t(14;18) translocation in their peripheral mononuclear cells, which may further contribute to lymphomagenesis [58]. Mutations in other oncogenes, such as c-myc, and regulators of apoptosis may be the important missing link to our understanding of lymphomagenesis in the setting of chronic HCV infection.

### Hepatitis C Virus and Monoclonal Gammopathy

Serum monoclonal gammopathy (MG) is an extrahepatic manifestation of HCV infection. A prospective study by Andreone et al. found 11% of monoclonal bands in HCV-positive patients versus 1% in HCV-negative patients, demonstrating a significant prevalence of monoclonal gammopathy in HCV-related liver disease [59]. Actually, a few HCV positive patients with MG can be considered affected by myeloma according to clinico-pathological characteristics; the US Veterans Affairs database evaluated by Giordano et al. revealed an increased risk for the development of Waldenstrom's macroglobulinemia (hazard ratio=2.76) with no associated increased risk of development of multiple myeloma in HCV patients [52].

### Therapy for HCV-related LPD

Recent studies support the rationale for the use of antiviral therapy in the context of low-grade HCV-positive NHL regardless of histological subtype [60][61][62].

Interestingly, in these studies, there is a clear correlation between HCV viral-load reduction and clinical response in LPD-infected patients. In intermediate and high-grade NHL, chemotherapy is usually necessary and antiviral treatment may serve as maintenance therapy after the completion of chemotherapy [63]. Because chemotherapy may lead to a substantial increase in the levels of viremia, cautious monitoring of the HCV RNA levels and transaminases is important; still, a consecutive exacerbation of the infection, making discontinuation of chemotherapy mandatory, is not unlikely to occure [63]. Regular monitoring of transaminases during treatment is essential because HCV-positive patients seem to experience increased short-term hepatic toxicity from chemotherapy. The use of rituximab either in monotherapy or in combination with antiviral treatment or chemotherapy or both appears promising [64][65], however more studies are needed to define the actual role of rituximab in treatment and recovery.

## 3. HCV-associated arthritis (HCV-AR)

Rheumatologic complications of HCV infection are common and include MC, vasculitis, Sjogren's syndrome, arthritis, and fibromyalgia [66][67]. There is a well-defined picture of arthritis associated with the presence of MC that consists of an intermittent mono- or oligoarticular, nondestructive arthritis affecting large- and medium-size joints [66][68]. Joint involvement is the most frequent extrahepatic manifestation of HCV infection. The HCV-associated rheumatic manifestation varies from 2% to 23%, depending on the geographic region and the design of the studies [69][70][71][72][73]. HCV-AR commonly presents as a rheumatoid-like, symmetrical polyarthritis (SP) involving mainly small joints or less commonly as intermittent mono- or oligoarthritis in large joints (IMO[74][75]. The different diagnosis between SP and other polyarthritides, especially rheumatoid arthritis (RA), can be a clinical challenge [68][72]. HCV-AR is similar to RA, but it usually runs a relatively benign course that, in contrast to true RA, is typically nondeforming and is not associated with articular bony erosions. Furthermore, unlike classic RA, ESR is elevated only in about half of the patients, and subcutaneous nodules are absent [76]. In the diagnosis; SP frequently meets American College of Rheumatology's classification criteria for RA. However, anticyclic citrullinated peptide antibodies (anti-CCP) provide an important clue in distinguishing RA from HCV-AR because anti-CCP is considered to be specific for RA [77][78]. Positive HCV antibody and HCV RNA, as well as the absence of bony erosions and subcutaneous nodules may be useful in distinguishing between HCV-related arthritis and RA. Risk factors for HCV infection such as transfusion and IV drug abuse or a history of hepatitis should be included in the history of present illness of any patient with polyarthritis [79]. In such patients serologic studies for hepatitis C should be performed [79]. Table 1 may provide help in these differentiations.

**Table 1:** Comparison between HCV-associated arthritis and rheumatoid arthritis

	**HCV-associated arthritis**	**Rheumatoid arthritis**
**Rheumatoid factor**	40-65%	50-85%
**ANA**	10%	30%
**Anti-CCPs**	Usually negative (2-4%)	Usually positive (55-90%)
**Cryoglobulin a**	40-55%	~1%
**Anti thyroid antibodies**	< 10%	1-32%
**Erosive arthritis**	Absent	Present

^a^ In patients with HCV-associated arthritis and MC: RF positivity (virtually 100%). Low C4 levels (50-85%)

### Treatment 

The optimal treatment for HCV-related arthritis has not yet been established and very little evidence about the treatment of HCV-AR has been reported in the literature [76]. Patients with HCV-AR in the absence of cryoglobulinemia have been treated successfully with NSAIDs, hydroxychloroquine, and low doses of prednisone [80]. The IMO subset is usually responsive to low doses of corticosteroids with or without hydroxychloroquine [33]. Administration of antiviral treatment (IFN) has not been associated with significant improvement, and in certain cases has exacerbated articular symptoms [80][81]. In a small number of resistant cases, methotrexate was successfully used without significant adverse effects on liver function. Nevertheless, extreme caution with very close monitoring of liver function and viremia levels is needed for patients starting such therapy [81]. Another study examined penicillamine as a potential treatment; however, despite an improvement of symptoms, penicillamine did not lead to a complete remission of the disease, and there is no information yet in the literature about the safety of this therapy [82].

In addition, new data has emerged about the role of Cyclosporine in suppressing HCV replication by targeting the cyclophilin B protein, which interacts with the C-terminal region of NS5B and appears to stimulate the RNA binding activity involved in HCV RNA replication [83]. These favorable results are predominant for HCV genotype 1b and 4a [84][85]. Because CsA is currently administered to treat several autoimmune disorders including inflammatory joint diseases [86][87], it may have a potential role in HCV-AR therapy as well. Anti-TNF therapy for RA in the setting of HCV appears to be safe and well tolerated, without apparent influence on the underlying HCV infection; however, the usually nonaggressive course of HCV-related arthritis does not justify the therapeutic use of anti-TNF [88]. Recently, a trial evaluated the safety and efficacy of Etanercept in a small group of HCV-AR. Although Etanercept has been reported to be safe, its efficacy does not seem promising, especially given that it produced lower results than those obtained in patients with RA [89]. The treatment of RA in patients with coexisting HCV infection is also problematic. First-line disease-modifying drugs such as Methotrexate and Leflunomide are potentially hepatotoxic and should be used with extreme caution [90][91]. In mild cases, Hydroxychloroquine can be tried first with and without low doses of Prednisone (<7.5 mg/d [92]. Anti-TNFα has also been used in patients with HCV infection without significant short-term side effects [91]. Once the diagnosis of HCV-AR is made, combination therapy with IFN-alpha (IFNα) and Ribavirin should be initiated. The use of antiviral drugs shows positive results, but IFNα can worsen autoimmune disorders [80]. Low-dose oral corticosteroids, nonsteroidal anti-inflammatory drugs, Hydroxychloroquine, or Sulfasalazine can be used in addition to the antiviral therapy to control arthritis-related symptoms [68][80].

## 4. Glomerulonephritis (GN)

**Figure 2:**
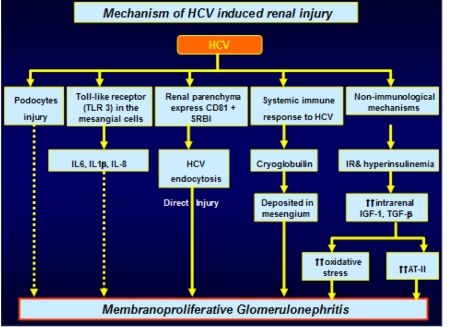
Mechanisms of HCV-induced renal injury

The association between HCV infection and renal disease is well established. Recent information at a population-based level has shown a significant link between HCV seropositivity and an increased risk for developing end-stage renal disease (ESRD; [93]. The mechanisms of HCV-induced kidney injury are summarized in figure 2. The common presentation of HCV-associated GN is proteinuria and microscopic hematuria with mild to moderate renal insufficiency. The majority of patients develop hypertension, often severe and difficult to control, and it may manifest acutely as oliguric-acute renal failure in 5% of cases [94]. The course of HCV-associated GN is characterized by remission and phases of relapse. The long-term outcome of HCV-associated nephropathies remains ill-defined. However, the overall prognosis for patients with HCV-associated GN is poor [93]. Patients with HCV infection and membranoproliferative glomerulonephritis (MPGN) tend to experience recurrences of both conditions following liver transplantation, and they have lower survival rates than do HCV-infected patients who do not have cryoglobulinemia or renal disease [95]. In all patients with proteinuria, a reduction in HCV RNA clearance has been documented at the end of IFN therapy [96]. The diagnosis of HCV-related MPGN is made by positive tests for serum HCV antibodies and HCV RNA. ALT levels are increased in 70% of patients, and the majority have low serum concentrations of complementary components (C1q, C4, and C3; [94]. Hepatitis C virus-associated posttransplant glomerulopathies, both recurrent and de novo, can occur in kidney-transplant recipients with HCV infection, especially in patients with higher HCV viral loads [97]. Another study found the prevalence of proteinuria to be greater among HCV-positive kidney-transplant recipients [98]. Membranoproliferative glomerulonephritis type I, focal and segmental glomerulosclerosis, minimal-change disease, membranous nephropathy, renal thrombotic microangiopathy, and mixed essential cryoglobulinemia in kidney transplant recipients have all been associated with HCV infection (Table 2) [97][98].

**Table 2:** Glomerular disease associated with HCV

**Common types**	**Less common types**
**MPGN, with cryoglobulinemia**	MPGN without cryoglobulinemia
	IgA nephropathy
	Postinfectious glomerulonephritis
	Membranous nephropathy
	Thrombotic microangiopathies
	Focal and segmental glomerulosclerosis
	Fibrillary or immunotactoid glomerulopathy.

### Treatment

For all patients, the decision to initiate treatment should be made after weighing the possible benefits and harms of therapy. The recommended therapeutic strategy depends on the severity of the kidney disease. For all patients, the same renoprotective measures (blood-pressure-lowering drugs and antiprotinuric agents) commonly used in patients with chronic nephropathies should be used in patients with HCV-associated GN. Diuretics, lipid-lowering agents, and angiotensin-converting enzyme inhibitors or angiotensin receptor blockers are potentially beneficial in patients with HCV-associated GN [99]. In a recent meta-analysis of clinical, controlled trials of the two treatments (antiviral versus immunosuppressive) described for HCV-related GN, the majority of patients had cryoglobulinemic GN [96]. The primary endpoint was the frequency of patients with a reduction of proteinuria (return of proteinuria to normal or a decrease of at least 50%) by the end of therapy. Pooling the results from this study demonstrated that proteinuria decreased more commonly after standard IFN-doses than with corticosteroid therapy and the OR was 3.86 (95% CI, 1.44; 10.33; P=0.007); however, both treatments failed to improve renal function. The authors concluded that the antiviral therapies were more effective than immunosuppressive therapy in lowering proteinuria levels in patients with HCV-related GN, at least in the short term. Therefore, the first-line treatment for patients with mild to moderate clinical and histological kidney damage is antiviral therapy. In case of severe renal involvement (nephrotic syndrome, nephritic syndrome, progressive renal failure, or a high activity score of glomerulonephritis on light microscopy), the initial treatment may consist of sequential administration of immunosuppressive therapies (plasmapheresis, corticosteroids, and cyclophosphamide). Caution should be taken using immunosuppressive therapy in patients with HCV-associated GN because of a concern regarding viral replication [100]. For patients unresponsive to steroid and immunosuppressive therapy, preliminary data support the use of rituximab for the treatment of HCV-associated GN [101]. This is a human-mouse chimeric monoclonal antibody that selectively depletes B-cell by binding to CD20 cell surface antigen [102]. It has been suggested that rituximab has a marked antiproteinuric effect through interference with monoclonal IgM production, cryoglobulin synthesis, and renal deposition of immune complexes (ICs). Recent data notes that Rituximab combined with Peg-IFNα/ribavirin is well-tolerated and more effective than Peg-IFNα/ribavirin in HCV-MC. In a recent prospective cohort study of 38 HCV-MC patients who received a combination of Rituximab (375mg/m² once a week for 1 month) followed by weekly Peg-IFNα (2a, 180mcg or 2b, 1.5mcg/kg) plus ribavirin (600-1,200 mg) daily for 48 weeks were compared to 55 HCV-MC patients who received the same Peg-IFNα and ribavirin treatments. Compared with Peg-IFNα and ribavirin, patients who received rituximab plus Peg-IFNα and ribavirin had a shorter time to clinical remission, better renal-response rates, and a high tolerance for treatment with no worsening of HCV RNA under rituximab [103]. Another very recent, long-term trial confirmed these results, which may last for over 3 years [104].

## 5. HCV and Thrombocytopenia

Several studies have shown that thrombocytopenia is frequently observed in patients with chronic hepatitis C infection [69][105][106][107][108], and a variety of pathogenic mechanisms that are implicated in this abnormal finding are portal hypertension and hypersplenism in the cirrhosis stage, autoimmune reaction to platelets, and direct infection of platelet and megakaryocytes by HCV infection. This may be a sign of extrahepatic manifestation of chronic hepatitis C [109]. In cirrhotic patients, sequestration of platelets in the enlarged spleen secondary to portal hypertension can cause thrombocytopenia [105]. However, thrombocytopenia also occurs in patients with chronic hepatitis C without cirrhosis. Another mechanism is autoimmune reaction to platelets [110][111]. Some reports indicated that HCV infection may reflect the expression of platelets-associated immunoglobulin G(PAIgG), which can lead to platelets destruction by the reticulo-endothelial system [112][113]. In addition, several studies have suggested that HCV may have a direct pathogenic role in the process leading to thrombocytopenia [105][114]. The incidence of mild thrombocytopenia (defined as a platelet count under 150,000/μl) is between 41% and 50% in patients with HCV infection, whereas severe thrombocytopenia (defined as a platelet count under 50,000/μl) is less common [105][115].

## 6. Cutaneous Manifestations of HCV

In addition to MC-related purpura, HCV infection also has been associated with several cutaneous disorders as noted below.

### Pruritus

Pruritus is a presenting symptom in 20% of patients [116]. Although, the pathogenesis is uncertain, both peripheral (increased plasma level of bile salts) and central mechanisms (increased plasma level of opioids) have been proposed [116]. The combination of both bile-salt-lowering and opioid-antagonist strategies appears reasonable in the management of pruritus of cholestasis; treatment options include topical antipruritics, systemic antihistamines, rifampin, naloxone or naltrexone, and ultraviolet B phototherapy [116].

### Porphyria Cutanea Tarda (PCT)

**Figure 3:**
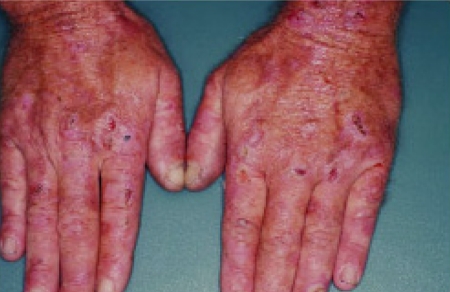
Erosions, crust, and blisters are evident on the hands of this patient with PCT.

PCT is a photosensitivity disorder caused by a decrease in functional uropophryinogen decarboxylase (UROD) and an increase in circulating porphyrins. The prevalence of HCV infection in patients with porphyria is high, ranging from 40% to 50% (Figure 3) [117]. HCV does not seem to induce alteration of porphyrin metabolism, although it may induce the disease in genetically predisposed individuals. Meanwhile, some authors suggest that PCT might be related to HCV-induced hepatic iron overload [117]. The highest rates of PCT have been observed in patients with HCV-related liver cirrhosis, suggesting that cirrhosis may play a role in its development. Antiviral therapy seems to ameliorate cutaneous lesions, but there is still no randomized clinical trial [117].

### Lichen Planus (LP)

**Figure 4.**
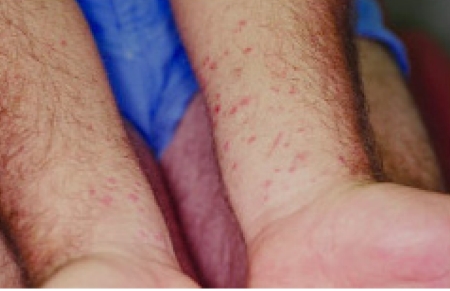
The polygonal purple papules of LP are evident on the forearms of a patient with HCV.

HCV has been implicated in triggering LP [118]. In a recent meta-analysis, LP patients were observed to have significantly higher risk of being HCV seropositive (odds ratio 4.85; 95% CI 3.58-6.56) than controls. A similar odds ratio of having lichen planus was found among HCV patients (4.47; 95% CI 1.84-10.86). Subanalyses indicated that the variability in the association between HCV and lichen planus seemed only partial and dependent on geographic effect Studies. [118]. Data from Egypt reveal that the prevalence of LP among CHC patients is around 4% [119]. Data suggest that skin and mucosal lesions may be caused by direct action of the virus or immunological response, especially when erosive oral lesions are present, and recently HCV-induced insulin resistance has been implicated in the pathogenesis of LP [118][120]. The skin and the oral cavity are easy to observe, so the presence of LP can be potentially used as a potential marker of HCV in asymptomatic patients (Figure 4) [121]).

### Treatment

LP is associated with a variable response to IFN treatment, and both improvement and exacerbation of symptoms have been reported. The uses of steroids, either topical or injectable, are also used in managing the symptoms [120].

## Link between HCV and other clinical disorders (7. Sicca syndrome)

Sjögren's syndrome (SS) is an autoimmune disease that involves the exocrine glands and generally induces xerostomia and xeroftalmia (sicca syndrome) due to an involvement of salivary and lachrymal glands in the presence of immunological alterations represented by antinuclear autoantibodies and antiepithelial neutrophil-activating peptide (ANA, SSA/Ro, SSB/La). In the absence of an associated systemic autoimmune disease, patients with similar manifestations can be defined as affected by primary SS. The clinical spectrum of the syndrome ranges from an organ-specific autoimmune disease to a systemic process with different manifestations. SS is recognized in large portions of MC patients [122][123], and was also observed in CHC patients by Haddad et al. in 1992 [124]. In a recent cohort from Egypt, SS's prevalence was reported to be around 9% [73]. This syndrome closely resembles primary SS; however, it typically lacks ANA, SSA/Ro, and SSB/La. The pathogenic role of HCV infection in SS remains an issue of debate [125]. It has been proposed that HCV infection is a criterion to rule out a primary diagnosis of SS, especially if cryoglobulinemia and hypocomplementemia are present and anti-SSA/Ro antibodies are absent [126][127].

## 8. Idiopathic pulmonary fibrosis (IPF)

A pathogenic link between HCV infection and IPF has been suggested by the higher frequency of HCV markers in patients with IPF [128][129]. Whether CHC is linked to pulmonary fibrosis directly or indirectly through underlying cryoglobulinaemia and vasculitis remains issue of discussion [130].

## 9. Cardiomyopathy and atherosclerosis

A causal relationship with HCV infection has been suspected for several myocardial impairments, including dilated cardiomyopathy, hypertrophic cardiomyopathy, and chronic myocarditis. The pathogeneses of these HCV-associated myocardial impairments probably rely on autoimmune phenomena with the particular involvement of the human major histocompatibility (MHC) class II antigen [131]. Moreover, the recent determination of a significantly higher prevalence of carotid or coronary artery atherosclerosis in patients with HCV infection [132][133] is noteworthy. The recent finding of HCV RNA sequences in plaque tissue strongly suggests an active local infection. This in turn makes it conceivable that the virus may exert local action in carotid atherosclerosis [134].

## 10. Neuropathy 

HCV-neuropathy presents with peripheral neuropathy that involves the legs and is typically very painful, with progression to muscle weakness in some patients. The disorder results from immune-complex deposits within the vasa nervorum of the peripheral nerves leading to vasculitis [135]. Antiviral therapy should be applied cautiously in the presence of neuropathy. Although cases of HCV-related peripheral neuropathy responsive to antiviral therapy with IFNα and ribavirin have been described [136], several authors have reported an aggravation of pre-existing MC-related neuropathy or even de novo occurrence of demyelinating polyneuropathy during IFN-α or PEG-IFN-α treatment [137]. Therefore, it is presumable that genetic susceptibility and other idiosyncratic factors may influence the response of the HCV-associated neuropathy to IFNα, making close monitoring of the course of this EHM during IFNα treatment indispensable.

## 11. Thyroid and HCV

Autoimmune thyroid diseases (AITDs) are complex diseases that develop as a result of interactions between genetic, epigenetic, and environmental factors. Significant progress has been made in our understanding of the genetic and environmental triggers contributing to AITD. The high prevalence of AITDs has been reported in HCV-infected patients before and after IFNα therapy [138]. Two well-documented environmental triggers of AITD, HCV infection and IFNα therapy, should be given more attention in the field [139]. Chronic HCV infection has been shown to be associated with an increased incidence of clinical and subclinical autoimmune thyroiditis (i.e., the presence of thyroid antibodies in euthyroid subjects). The pattern of thyroid disorders observed in HCV infection is characterized by the presence of increased circulating antithyroid peroxidase antibodies (AbTPO) and an increased risk of hypothyroidism in AbTPO-positive subjects [109][140]. The Autoantibodies against internal organs such as the thyroid are common before therapy with IFNα [141]. In some cases, antibodies against IFN appear after IFN therapy with in HCV-infected patients [142]. Moreover, IFNα  therapy for chronic HCV infection is associated with subclinical or clinical thyroiditis in up to 40% of cases, which can be autoimmune or nonautoimmune thyroiditis. In some cases, IFN induced thyroiditis (IIT) in chronic HCV patients may result in severe symptomatology necessitating the discontinuation of therapy. Although the epidemiology and clinical presentation of HCV- and IFN-induced thyroiditis have been well-characterized, the mechanisms causing these conditions are still poorly understood.

### Treatment

In cases of symptomatic hyperthyroidism,discontinuation of therapy is recommended. In hypothyroidism, adding thyroid hormone can alleviate the symptoms, making it possible to continue therapy. Thyroid-function tests should be provided every 3 months during the therapy.
